# Multistage Framework for Automatic Face Mask Detection Using Deep Learning

**DOI:** 10.1155/2022/1500047

**Published:** 2022-08-11

**Authors:** Sowmya K. N, Rekha P. M, Trishala Kumari, Baru Debtera

**Affiliations:** ^1^Department of Information Science and Engineering, JSS Academy of Technical Education, Bangalore, India; ^2^Mercedes Benz Research and Development, Bengaluru, India; ^3^Department of Chemical Engineering, Addis Ababa Science and Technology University, Addis Ababa, Ethiopia

## Abstract

The whole world is fighting as one against a deadly virus. COVID-19 cases are upon us in waves, with subsequent waves turning out to be worse than the previous one. Scores of human lives are lost while the post-COVID-19 complications are on a rise. Monitoring the behaviour of people in public places and offices is necessary to mitigate the transmission of COVID-19 among humans. In this work, a low-cost, lightweight two-stage face mask detection model is proposed. In the first stage, the model checks if a face mask is worn. In the second stage, it detects if the mask is worn appropriately, by classifying and labelling them. The proposed models are trained to detect faces with and without masks for varied inputs such as images, recorded videos, and live streaming videos where it can efficiently detect multiple faces at once. The efficacy of the proposed approach is tested against conventional datasets as well as our proposed dataset, which includes no masks, surgical masks, and nonsurgical masks. In this work, multiple CNN models like MobileNetV2, ResNet50V2, and InceptionV3 have been considered for training and are evaluated based on transfer learning. We further rely on MobileNetV2 as the backbone model since it has an accuracy of 98.44%.

## 1. Introduction

COVID-19 has hit us hard on all fronts. With a total of 183+ million COVID-19 cases globally, no one can deny that a tiny virus can be deadly for the entire human population. The human costs range from financial and emotional to mental and are not limited by geography. Isolation, masks, and sanitisers have all become keywords. Additionally, gender-based violence has increased during this COVID-19 era. A lockdown imposed by any nation cannot continue arbitrarily for a long period since people from all ages and walks of life suffer a lot due to immobility, financial burdens, and so on. All the governments are taking preventive actions by vaccinating people and introducing social norms as preventative steps to stop the spread of the virus. Wearing a facial mask is the first step towards winning the fight against the COVID-19 virus along with vaccination. Each of us needs to wear a face mask properly to save ourselves against hospital bills and succumbing to COVID-19. To monitor individuals at the entrance of public places like malls, airports, offices, schools, colleges, and social gatherings, in this work, a transfer learning approach is adopted to detect whether a person is wearing a mask and further classify if the person is wearing the mask correctly by covering his nose and mouth completely. The proposed model is useful in places where there are too many people and tracking everyone manually is difficult. The proposed model is built on the concept of transfer learning.

Transfer learning is very popular in deep learning as it reuses the previously trained model for a new problem effectively with relatively small data. This proves to be helpful in the field of data science because in the real world, problems do not contain million data points with a label to train complex models. With transfer learning, one can try to apply the successful technique that one has learned in one task, to improve the performance of another. We transfer network-learned tools in task A to new task B. It helps to use the data labelled for the work it was originally trained for. Transfer learning has several benefits, but the biggest benefits are saving training time, better functioning neural networks, and not requiring lots of data. Transfer learning can be realised using pretrained models. A pretrained model is previously trained on a dataset and contains the weights and biases that represent the features of whichever dataset it was trained on. Learned features are often transferable to different data. As pretrained models, various CNN models can be used for object detection, identification, prediction, and classification.

The following is a list of the proposed work's significant contributions:

- The novel face mask detector approach not only detects faces wearing masks but also concentrates on detecting faces wearing all variants of the correct mask.
- All forms of masks (surgical, N95, and cloth masks) worn by people are included in the dataset. There are no faces that are biased toward a specific region or country.
- JSSFMD dataset is comprised of people with correct and incorrect face mask.
- Face detection at possible multiple angles, when there is fast head movement, and also occluded face detection under various illumination conditions have all been considered in the proposed work.
- This work effectively processes a real-time video feed with a high frame rate in a considerable time.
- Face mask recognition is considered for static images, recorded videos, and real-time streaming videos, which might be useful in tracking persons who do not obey the mandatory rule of wearing a face mask.
- The proposed approach is a low-power model and is fast and easy to use in devices with limited computing facilities.

The organisation for the rest of the paper is as follows.  Section 2 reviews prevalent works related to face recognition and face mask detection.  Section 3 describes the proposed transfer learning methodology adopted in detail.  Section 4 provides the analysis of experiments conducted with pretrained models considered and illustrates the implementation details, and Section 5 depicts the experimental results obtained followed by Section 6 which provides the conclusion of the proposed work and future scope.

## 2. Literature Review

The rapid adoption of deep learning techniques, particularly deep convolutional neural networks (CNN) as a backbone with computer vision techniques for object detection, identification, and classification, has helped to resolve multiple problems associated with pattern recognition for real-world problems. In the field of computer vision, supervised or unsupervised based learning models have been considered to equip the work of detecting objects in images and videos. In the face mask detection problem, at the outset, a face object is segmented and then the mask over the face. Object detection is one of the important features of computer vision. It has various applications like face detection, pedestrian detection, and classification [1]. Object detection with binary classifiers can improve object detection in video frames [2]. For the detection of an image with a face mask, the face needs to be detected first. Then, face detection models can be crossed with other methods for specified object detection like a mask on the face. ElMaghraby et al. proposed to use a hybrid of Viola-Jones and skin detection techniques to forthwith low-resolution face detection in static images and frames of the video [3]. Hybrid methods of advanced deep learning and classical machine learning are used to detect face masks [4]. Multiple face detection models like the RetinaFace model [5], MultiTask Cascaded Convolutional Neural Network (MTCNN) [6], Haar Cascade [7], and Deep Neural Network (DNN) [8] are used to detect faces. Royet al. proposed a mask detection model for monitoring mask wear by humans in real time. Object detection algorithms are like single-shot detector (SSD), Faster R-CNN, YOLOv3, and YOLOv3Tiny [9]. The YOLOV3 object detection method was adopted by Bhuiyan et al. along with DarkNet53 [10]. Addagarla et al. proposed methods for the identification and classification of real-time multiscale facial masks by using the techniques NASNetMobile, ResNet, SSD300, and YoloV3 as deep convolutional based networks for object detection and classification tasks [11]. SSD is based on spatial separable convolution, and to strengthen the deep features, Feature Enhancement Module (FEM) has been used for real-time face mask detection [12]. Rao et al. proposed a new CNN architecture called M-CNN to ascertain if a mask is worn by an individual. They built the sequential layer in the CNN model [13].

With the increase in training models in recent times, transfer learning is taking an important place in computer vision applications. Transfer learning utilises the pretrained tasks to train new tasks. For making the CNN model for face mask detection, Chowdary et al. used InceptionV3 as the backbone model [14]. MobileNetV2 [15], a light model, is used as a base model for various models in transfer learning. Razavi et al. in their face mask detection system adopted multiple Faster R-CNN object detection models like Inception-ResNetv2, ResNet152 V1, SSD ResNet50 V1, and SSD MobileNetV1 and inferred Inception-ResNetv2 as the best model for detecting accurate face masks [16]. Popular datasets used by multiple researchers in the area of face mask detection include SMFD [4, 14], RMFRD [5], Larxel [5], and LFW [4]. Real-world Masked Face Recognition Dataset (RMFRD) contains 5000 faces with masks and 90,000 unmasked faces. Simulated Masked Face Dataset (SMFD) contains 1570 images. In this, 785 represent simulated faces with masks and 785 faces with no masks [4, 14]. Labelled Faces in the Wild (LFW) consists of 13,000 faces with masks. Roy et al. have created a Moxa3K dataset that contains 3000 images of masked and unmasked faces [9]. Ge et al. introduced a dataset MAFA consisting of 30,811 normal images and 35,806 faces with masks[17, 18]. To avoid COVID-19, people should wear masks as well as maintaining proper social distance [15, 19, 20]. Face mask detection techniques can be used in various places like places of public health care, malls, smart city networks, supermarkets, construction sites, etc. This model can also be implemented using different hardware like CCTV [21] and raspberry pi [20]. Meta-analytic surveys of face masks have been elaborated in [22].

To solve the problem of detecting a face mask, a deep-learning model based on transfer learning trained on a highly tuned and customised face mask dataset, compatible with video surveillance is being proposed and discussed in detail in the next section.

## 3. Methodology

The importance of face masks in preventing COVID-19 has mandated mankind to garb it appropriately over the nose and mouth to prevent infections. Governing bodies require technical assistance to identify the people who violate COVID-19 norms. Our proposed face-mask detection system will assist them to achieve the same. We propose a model hinged with advanced deep learning and transfer learning concepts of machine learning to classify faces with a safety mask and ones without them. In the second stage, the faces detected with masks are verified to check if mask-wearing is correct or incorrect. Transfer learning-oriented neural network models based on deep learning have been considered for classification to achieve better performance for all pretrained models—MobilenetV2, Resenet50V2, and InceptionV3—are considered in our work. They have been trained with the ImageNet dataset [23] which contains more than 14 million images.

### 3.1. Proposed Architecture

**Figure 1:**
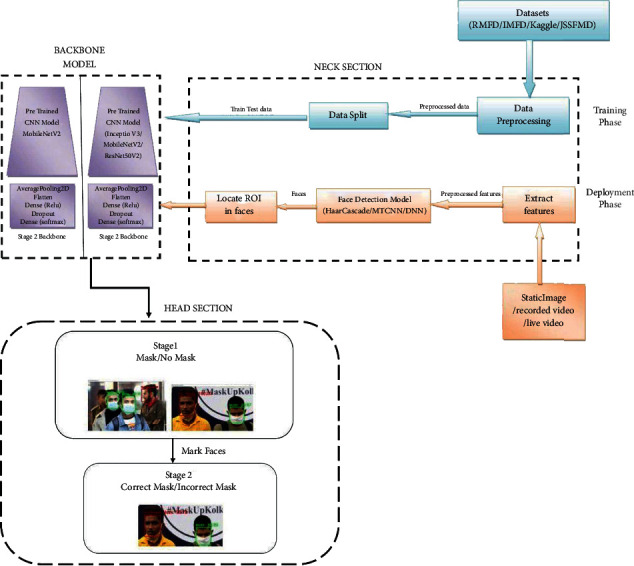
Proposed multimodel face-mask detection architecture.

The proposed solution architecture is a two-stage architecture based on the CNN model as its backbone as shown in Figure 1. The first stage is to detect all the faces with or without a mask. The second stage verifies if the masked person has worn the mask properly or not. The proposed model is trained using image samples collected from Kaggle [24], RMFD [1], IMFD [25], and our JSSFMD [26] datasets and search engine results. Deep and transfer learning is employed to train the proposed model. Performance is measured for 3 pretrained models, MobileNetV2, ResNet50V2, and InceptionV3, and the one with the best accuracy and time complexity is selected as the base model for backbone. Input to the proposed model can include static images, recorded videos, and live video feeds through a webcam or camera. The input image or frames from the video extracted are preprocessed and then fed to the face detection model. Haar Cascade, MTCNN, and DNN face detection models have been considered in this work. The facial representative results obtained are then fed to a training model for classifying the faces as “With Mask” and “Without Mask” in stage 1, and in stage 2, “With Mask” faces are further evaluated to verify the correctness of mask worn as “Correct Mask” and “Incorrect Mask” based on the input image or video feed.

#### 3.1.1. Processing Using the Convolution Neural Network Model

After preprocessing, the input samples must be trained. The following CNN models were investigated for this purpose.

InceptionV3 model was built to minimise the error rate of Deep Neural Networks by Szegedy et al. InceptionV3 is Google version 3 in the deep learning convolutional architecture series with a 48-layer network. It has been pretrained with extensive classes and the final layer will utilise the bottleneck value to predict results that are compared with the actual labels. The final layer's weight is calculated using the backpropagation approach. Additionally, 5 layers have been considered for fine-tuning utilising the transfer learning approach.

ResNet model was introduced in 2015 and stands for residual network. Its main aim is to resolve a complex problem by adding additional layers that are stacked into Deep Neural Networks, leading to improved accuracy and performance. The concept behind adding more layers is that these layers would learn increasingly complicated features as time goes on. There is a direct connection that skips some levels in the middle. The core of residual blocks is those links that were skipped and is known as the skip connection. ResNet50 is a convolution neural network that is 50 layers deep. Out of 50 layers, 48 layers are convolution layers along with MaxPool and average pool layers. ResNet50 has a very dense neural network architecture with 4 stages. ResNet50 version 2 focuses on preactivating weight layers rather than postactivating them. ResNet50V2 can detect and classify complex objects but it is too expensive in terms of computational memory.

MobileNet model was introduced with a feature of depth-wise separable convolution. In addition to depth-wise separable convolution, MobileNetV2 has a linear bottleneck and inverted residual feature. MobileNetV2 is a small, low-cost, low-power model. It is fast, easy to use, and suitable for areas with limited computing power. It works best on CPUs instead of more expensive GPUs that utilise more resources. The MobileNetV2 architecture contains the initial fully convolutional layer with 32 filters, followed by 19 residual bottleneck layers. They use ReLU6 as nonlinearity due to its robustness when used in the low precision computation.

### 3.2. Dataset Elucidation

**Figure 2:**
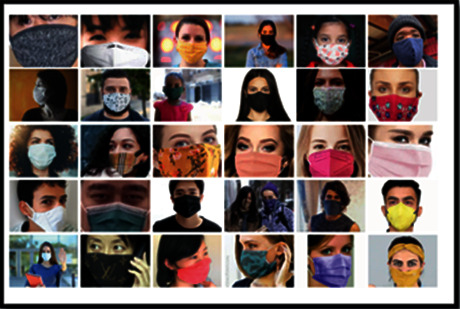
Sample images from datasets with the mask.

Benchmark datasets have been used in our work for face mask detection. Our dataset comprises 14,351 samples that are grouped into two sets for stage 1 and stage 2, respectively. The dataset which is used to train stage 1 contains a total of 7830 images. In this, 2500 images comprising people with face mask have been taken from the Kaggle dataset [24], and 800 images from the real-world mask face dataset (RMFD) [1]. We find that in literature most of the images were biassed toward Asian faces. Henceforth, we collected 500 images from search engine results and 120 images by taking photo feeds. Very few images are synthetic in nature. All types of face masks including surgical, N95, and cloth masks worn by people around the world are present in our dataset. They are classified into two classes—mask and without a mask. 3920 images belong to the class “with_mask” and 3910 images belong to the “without_mask” category. Occluded faces are included in the “without_mask” category to avoid confusion have considering these faces as “with_mask” faces.  Figure 2 shows the sample of faces with masks of all types considered. The train and test split ratio considered is 0.2, so the proposed model has 6256 images for training and 1565 images for testing purposes in stage 1.

**Figure 3:**
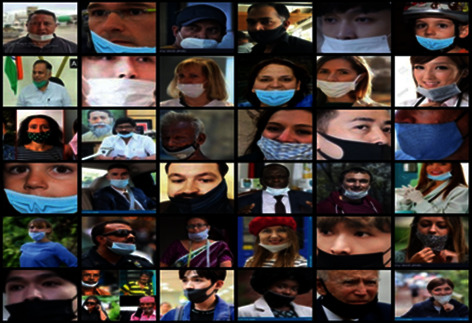
Sample images from datasets with incorrect mask.

In our daily lives, we have seen people wearing face masks below their nose or chin and sometimes on the neck, citing invalid reasons. The attitude of such people is a primary concern and identifying such covidiots is an added task for the authorities. The dataset used in stage 2 consists of 6521 images. In this, 2601 images are with incorrect_mask class, 1901 out of which were taken from the Incorrectly Masked Face Dataset (IMFD) [25]. All the images in IMFD are artificially created. 700 images were collected from different platforms on the Internet where people wore the masks incorrectly. The remaining 3650 images belong to the “Correct Mask” category. In Figure 3, the sample images from the dataset with people wearing incorrect masks like wearing masks below the nose, lip, and chin are shown.

All the dataset samples used in both stages are consolidated in JSSFMD [26] and are classified under Set1 and Set2 categories.

### 3.3. Pseudocode for Face Mask Detection

The steps adopted in training and deployment of the proposed face-mask identification using a deep learning model for effective surveillance are discussed (Algorithm 1).

The steps specified in the algorithm are elaborated in Experimental Setup and Implementation, respectively.

## 4. Experimental Setup and Implementation

The proposed architecture has been implemented with a system having an Intel i5 core processor (4.1 GHz), 16 GB RAM, and a Radeon Graphics card. The Jupyter Notebook IDE with a Python 3.8 kernel has been selected for training the proposed 3 CNN models. For face mask detection, MobileNetV2, InceptionV3, and ResNet50V2 models are trained to classify input data as masked or unmasked in stage 1 and correct mask or incorrect mask in stage 2, respectively. The Keras framework from the TensorFlow library is used to train the models using a transfer learning approach. Initially, these models are trained using pretrained ImageNet weights.

### 4.1. Data Collection Loading

Benchmark face masked dataset images RMFD [1], IMFD [25], and Kaggle [24], along with the results obtained from search engine results, are collected in the first phase [26]. The collection includes data on people who wore masks, those who wore the wrong masks, and those who did not. The TensorFlow deep learning library, along with Keras, is used to load and read the dataset. This model has two labels for the first stage: “with mask” and “without a mask.” In the second stage, the dataset labels are “Correct Mask” and “Incorrect Mask.”

### 4.2. Data Preprocessing

The input image frame of a video is preprocessed to remove distortion and redundancy. During data processing, the given raw data is modified into a useable format. The steps involved in preprocessing data include resizing the image and converting the image into a single dimensional array. Resizing an image is required to set a base size and improving the effectiveness of the model. The dataset images are resized to 224 × 224 pixels. The next step is to represent these images into an array as per the model requirement. The data is augmented using the Keras preprocessing class that allows us to perform normalisation and random transformation operations on training images. Random rescaling, horizontal flips, brightness/contrast/colour changes, and random cropping are all considered here.

### 4.3. Splitting of the Dataset

After preprocessing of the dataset, the data is split into two groups: training data and testing data in the ratio of 0.2. 80% of the total dataset is used for training and 20% for testing. The first stage model has 6256 images for training and 1565 images for testing. In stage 2, the model has 5224 images for training and 1306 images for testing after the split.

### 4.4. Face Detection Model

Face detection models are loaded to detect human faces in video and images. The face detection models considered in this work include Haar Cascade, MTCNN, and DNN models.

Haar Cascades: Haar features are extracted from images and passed through a cascade of classifiers [20]. These cascades are available in the OpenCV library along with the XML files.

MTCNN: multitask cascade neural network (MTCNN) adopts three network layers in a cascading approach. P-Net locates the possible candidates with faces from image pyramids. R-Net refines them further by removing insignificant bounding boxes, while O-Net locates landmarks over the detected faces in the final stage. This model effectively reduces the false positive outputs at a faster rate.

DNN: it adopts the single-shot-multibox detection approach. This compositional Caffe model uses ResNet10 architecture as its backbone.

In stage 1, Haar Cascade and MTCNN pretrained face detection models are considered. The results of Haar Cascade are unsatisfactory since it is unable to recognise side faces and produces a large number of false positives. The MTCNN model chosen avoids false positives and correctly detects all of the faces in the image. Therefore, MTCNN is used as a face detection model for static images and recorded video. MTCNN output is a little slow for streaming videos since it gives frame-wise output. Therefore, the DNN face detection model is considered for live feeds. The DNN model gives output as a continuous stream. The final output of all models is tested for different angles of the face, head movement, occlusion of face, multiple lighting conditions, and frame rates. OpenCV's Caffe model from the DNN module is the best among the three.

### 4.5. Building the Model

The proposed deep learning model utilises the transfer-learning approach. It allows us to work efficiently and save time. The transfer-learning enabled models are initially pretrained with big datasets that include weights and biases that describe the initial dataset's properties. The characteristics are then applied to other data sets as required. In this work, MobileNetV2, ResNet50V2, and InceptionV3 models are utilised for constructing a base model for the backbone architecture. The base model accepts a 224 × 224 × 3 input image. The model backbone is trained using ImageNet [23] weights that have already been trained. The head model consists of various add-on layers like AveragePooling2D with the pool size of 7 × 7, flattening layer, dense layer with activation ReLU, dropout to reduce overfitting, and the last dense layer with the activation Softmax. Softmax function gives two probabilities that represent the classification of mask or no mask in the first stage and correct mask and incorrect mask in the second stage. Before training a complete backbone model, the base model is frozen and the weights are not changed further. Based on loss backpropagation, the Adam optimizer function is utilised to adjust the learning rate and weights. To find the loss in the proposed binary model, cross-entropy is estimated. All of the models have been trained for different epochs. Each model's loss and accuracy are continuously monitored. For the second stage in the proposed CNN model, the MobileNetV2 architecture is adopted for optimization purposes.

### 4.6. Performance Analysis of Proposed Models

Keras applications are available for a variety of classification tasks. They comprise deep learning models that come with pretrained weights. We employ three pretrained applications in this work and have modified CNN layers on top of them. Prediction, feature extraction, and fine-tuning tasks are executed with the chosen models. Convolutional neural networks like Inception, ResNet, and MobileNet are primarily used for image classification. Inception minimises the computing cost of Deep Neural Networks while achieving cutting-edge performance. The inception model rescales neural networks without an increase in their processing cost since the computational efficiency of a network decreases as it grows deeper. ResNet is related to computational accuracy, while Inception is associated with computational cost. Improved accuracy in computer vision problems is possible by creating deep and more sophisticated networks. Deep networks come at the additional cost of size and speed. MobileNet, a network for embedded vision applications and mobile devices, enables object detection on a computationally restricted platform in a real-world application.

**Figure 4:**
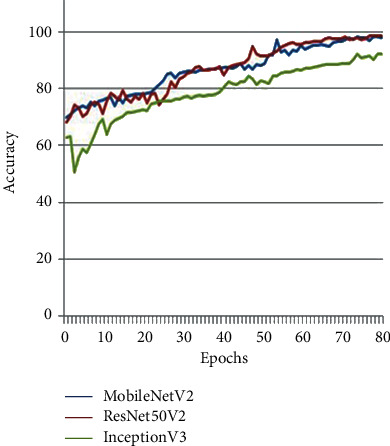
Accuracy achieved by MobileNetV2, ResNet50V2, and InceptionV3.

**Figure 5:**
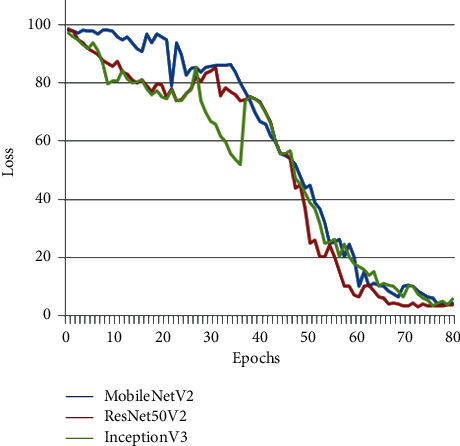
The loss plot that illustrates decrease of training data loss over increasing number of epochs.

Comparative analysis of the proposed models, MobileNetV2, ResNet50V2, and InceptionV3, in terms of “Accuracy” versus variant “Epochs” is shown in Figure 4. The graph depicts that the increasing number of epochs is proportional to increased accuracy.  Figure 5 indicates the loss plot illustrating the decrease of training data loss with an increased number of epochs.

**Figure 6:**
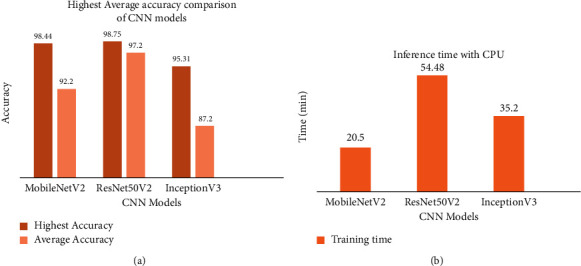
(a) Comparison of accuracy for CNN models. (b) Comparison of training time for CNN models.

Figures 6(a) and 6(b) demonstrate the inference times using the CPU after training the models for roughly 80 epochs to increase accuracy.

Experiments show that all three models have a reasonable level of accuracy (see Figure 6(a)). Due to its vast network, ResNet50V2 has the best accuracy of 98.75 percent, followed by MobileNetV2 (98.44 percent) and InceptionV3 (98.44 percent) (95.31 percent). ResNet50V2 has a high level of accuracy, but it takes a long time to train because of its complexity. In terms of time complexity, MobileNetV2 outperforms the other two models with optimal accuracy. As a result of this performance comparison, the model trained using the MobileNetV2 architecture is selected as the backbone model for stage 2.

## 5. Experimental Results

Experiments were conducted using MobileNetV2, InceptionV3, and ResNet50V2 models for face detection using Haar Cascade, MTCNN, and DNN models over the JSSFMD [26] dataset for training purposes. The pretrained models were then verified for variant inputs like images, recorded videos, and live video feeds. The results of the proposed transfer learning model adopted are discussed here.

**Figure 7:**
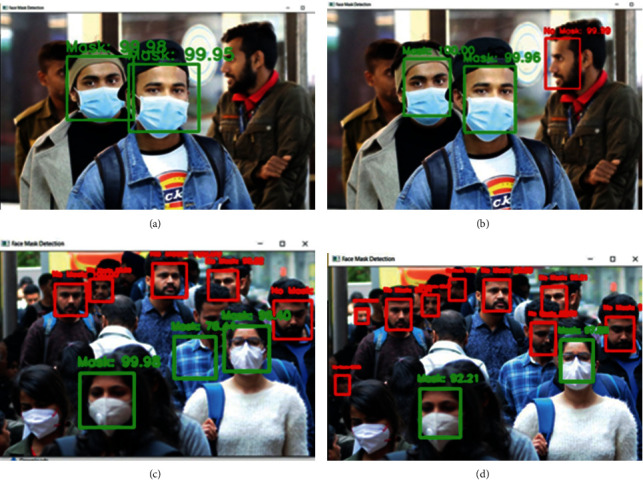
(a) Haar Cascade not able to detect side faces. (b) MTCNN able to detect side faces. (c) False positive with Haar Cascade. (d) No false positive with MTCNN.

Automatic verification of the proposed trained model is carried out for static images and recorded video inputs by detecting faces using Haar Cascade and MTCNN models. The MTCNN model generates better results when compared to the Haar Cascade face detection model as shown in Figure 7 for static images. The MTCNN model is good at detecting faces with orientation and avoids false positive results that are more likely to occur with the Haar Cascade as shown in Figures 7(a) and 7(c). MTCNN outperforms Haar Cascade in detecting multiple faces in an image as shown in Figures 7(b) and 7(d), respectively.

**Figure 8:**
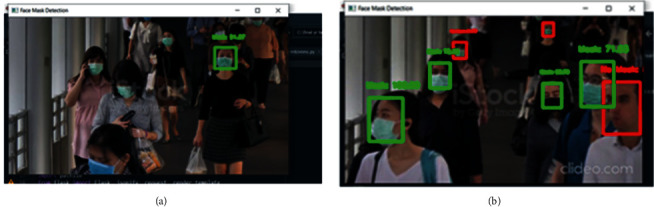
(a) Result with Haar Cascade on the recorded video. (b) Result with MTCNN on the recorded video.

MTCNN and Haar Cascade results indicating the number of faces detected for a frame in a recorded video are shown in Figures 8(a) and 8(b), respectively. MTCNN can detect more faces per second in one frame.

**Figure 9:**
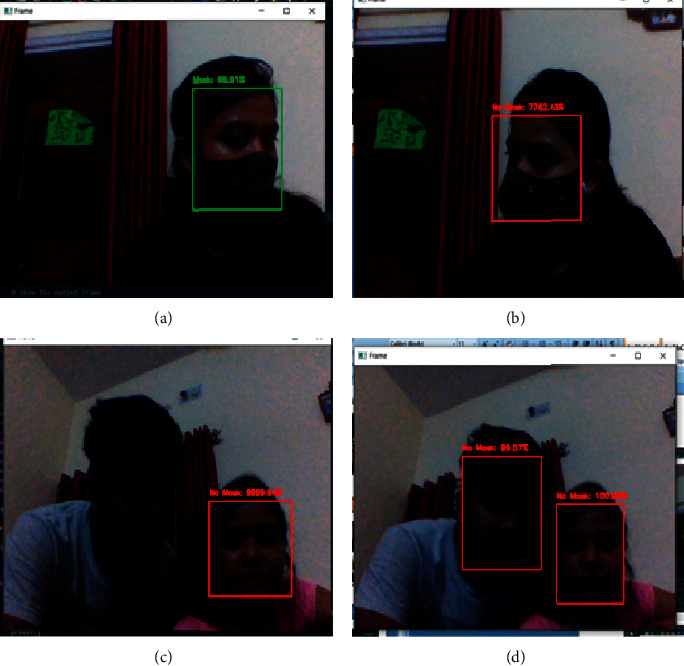
(a) Real-time DNN result for a side face. (b) Real-time MTCNN result for a side face. (c) Real-time MTCNN result in dim light. (d) Real-time DNN result in dim light.

MTCNN is best for static images and recorded videos but when implemented on real-time videos, results are not satisfactory. Real-time videos have their constraints like motion, blur, focus, frame transition, and jitter. To manage all these constraints, the DNN face detection model is adopted. It detects faces in all types of illumination conditions, side faces, and fast-moving faces in motion efficiently. Figures 9(a) and 9(b) show the comparison of results for a side angle face for a frame to a live video feed between the DNN and MTCNN model. Figures 9(c) and 9(d) represent the result for a low illumination scene.

**Figure 10:**
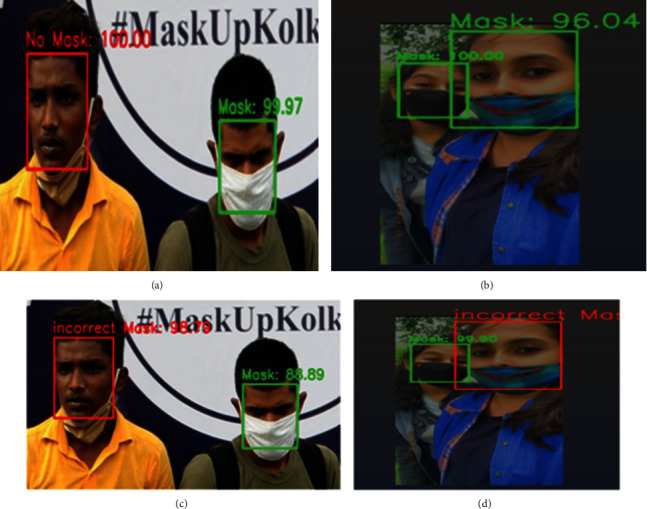
(a) Incorrect mask (below chin) identified as “no mask” in stage 1. (b) Mask below nose resulting as “mask” in stage 1. (c) Incorrect mask (below chin) in stage 2. (d) Incorrect mask (below nose) in stage 2.

In stage 2, verification of whether a mask is worn properly or not is considered using the MobileNetV2 model. In stage 1, either mask or no mask is detected as shown in Figures 10(a) and 10(b). In stage 2, validation of the face mask worn as correct or incorrect is found as shown in Figures 10(c) and 10(d).

**Figure 11:**
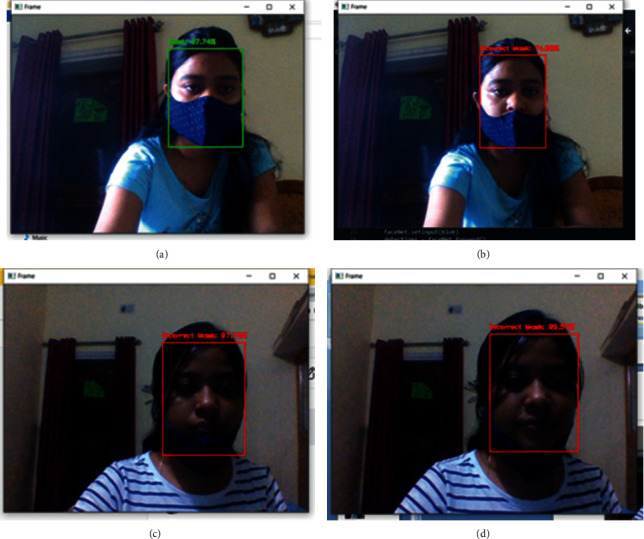
(a) Face with correct mask. (b) Face with incorrect mask (below nose). (c) Face with incorrect mask (below mouth). (d) Face with incorrect mask (below chin).

Results of the real-time video feeds for faces with correct and incorrect masks are shown in Figures 11(a)–11(d), respectively.

All three models—MobileNetV2, ResNet50V2, and InceptionV3—are considered during stage 1. MobileNetV2 is chosen for stage 2 since it outperforms the other two models on average. The proposed two-stage approach makes it efficient when compared to other solutions available.

## 6. Conclusion

Technical assistance to identify people not adhering to wearing a face mask is a necessity today to warn and take suitable action against them. Face masks help to contain the distance and volume of expiratory droplets that get dispersed when talking, breathing, and coughing by a COVID-19 subject. In this work, a transfer-learning approach for mask detection is adapted to detect COVID-19-norm violations in public places. The primary architectural backbone idea consists of MobileNetV2, MTCNN (Multi-Task Cascaded Convolutional Neural Networks), and DNN (Deep Neural Network) models that work effectively in both high and low computation scenarios. MobileNetV2 provides robust features to train the large dataset of images collected from Kaggle, RMFD, IMFD, Google search, and camera photo collection made available in the JSSFMD dataset. The proposed experimental framework has been used to test static images, recorded videos, and real-time videos. The MTCNN model gives decent outcomes for static images and videos. DNN model effectively detects faces in different orientations, occluded faces, and faces in multiple lighting conditions for real-time video. The technoworld we live in today requires automating the face mask detection process and our approach is a step towards it to benefit the society at large.

## Figures and Tables

**Algorithm 1:**
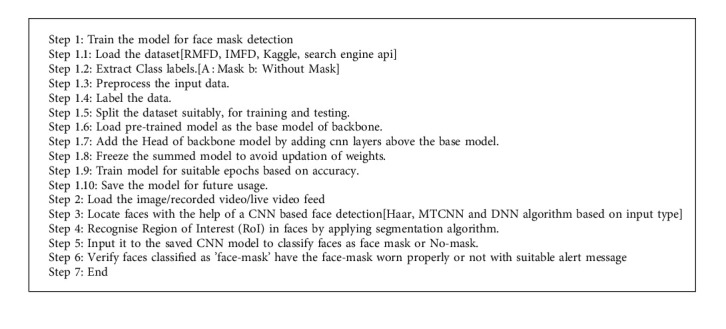
Face mask detector algorithm.

## Data Availability

The datasets used and/or analyzed during the current study are available from the corresponding author on reasonable request.
